# Clinical characteristics and MRI features of autoimmune glial fibrillary acidic protein astrocytopathy: a case series of 34 patients

**DOI:** 10.3389/fneur.2024.1375971

**Published:** 2024-03-22

**Authors:** Gaotan Ke, Si Jian, Tingxin Yang, Xu Zhao

**Affiliations:** Department of Radiology, Tongji Hospital, Tongji Medical College, Huazhong University of Science and Technology, Wuhan, China

**Keywords:** glial fibrillary acidic protein, astrocytopathy, hyponatremia, perivascular radial enhancement, modified Rankin scale score

## Abstract

**Objectives:**

To analyze the clinical and imaging characteristics of autoimmune glial fibrillary acidic protein astrocytopathy (GFAP-A).

**Methods:**

Forty-three patients diagnosed with GFAP-A between March 2017 and July 2023 were retrospectively recruited. The clinical characteristics and magnetic resonance imaging (MRI) features were collected.

**Results:**

Twenty-one patients (61.8%) had a fever and 16 (47.1%) had a headache. Five patients (14.7%) had coexisting neural autoantibodies and one patient (2.9%) had a coexisting neoplasm. The most common presentation was meningoencephalomyelitis (13/34, 38.3%), followed by meningoencephalitis (12/34, 35.3%). The other clinical manifestations included blurred visions (5/34, 14.7%) and peripheral nervous system involvement (4/34, 11.8%). Twenty-six patients (76.5%) had elevated nucleated cell count, predominantly lymphocytes (15/15, 100%), and 27 (79.4%) had elevated protein levels of cerebrospinal fluid. One-half (50%) of the patients presented with hyponatremia. A majority of the patients (30/33, 90.9%) exhibited abnormal hyperintense lesions on T2WI, which were often located in juxtacortical white matter (18/33, 54.5%), followed by periventricular white matter (16/33, 48.5%), basal ganglia (15/ 33, 45.5%), brainstem (11/33, 33.3%), and thalamic lesions (9/33, 27.3%). Twenty-four patients (72.7%) had abnormal brain enhancement, with supratentorial leptomeningeal enhancement being the most frequent enhancement pattern (15/33, 45.5%), followed by linear perivascular radial enhancement (14/33, 42.4%). Nineteen patients (70.4%) had hyperintense intramedullary spinal cord lesions, with long segments (15/27, 55.6%) and transverse lesions (14/27, 51.9%) being the most frequent lesions. Most cases were sensitive to immunotherapy, such as glucocorticoids, intravenous immunoglobulin, and tacrolimus, with three patients (8.8%) experiencing relapses. Patients with brainstem lesions had higher onset modified Rankin scale scores and were more prone to intensive care unit admissions. Linear perivascular radial enhancement was positively associated with poor prognosis (*p* < 0.05).

**Conclusion:**

GFAP-A presented with meningoencephalomyelitis and meningoencephalitis. The brain lesions were often located in juxtacortical white matter, periventricular white matter, basal ganglia, brainstem, and thalamus. Long segments and transverse were the most frequent spine lesions. Leptomeningeal enhancement was the most frequent enhancement pattern, followed by linear perivascular radial enhancement, which may provide new insight into the differential diagnosis of GFAP-A.

## Introduction

Glial fibrillary acidic protein (GFAP) is the main intermediate filament protein in mature astrocytes with multiple biological functions, including maintaining astrocyte morphological stability, participating in blood–brain barrier formation, and regulating synaptic function (1). GFAP is mainly expressed in mature astrocytes in the gray matter, white matter, cerebellum, subventricular and subgranular regions, Müller cells in the retina, and is also expressed in peripheral Schwann cells (2, 3). Autoimmune glial fibrillary acidic protein astrocytopathy (GFAP-A) — first described in 2016 by Fang et al. — has been characterized as a spectrum of steroid-responsive autoimmune inflammatory central nervous system (CNS) disorders (4). Detection of GFAP-immunoglobulin G (GFAP-IgG) in cerebrospinal fluid (CSF) is a biomarker of GFAP-A. This disorder typically manifests as encephalitis, meningitis, myelitis, optic neuritis, or a combination of the above. The characteristic imaging feature is linear perivascular radial enhancement in the white matter extending radially outward from the ventricles on magnetic resonance imaging (MRI) (4–6). No definite diagnostic criteria have been established yet (7). Although several GFAP-A case series have been reported, their clinical and imaging heterogeneity has posed great challenges to clinical diagnosis and treatment. Therefore, the current study retrospectively analyzed the clinical and MRI features of 34 GFAP-A patients, which may provide new insights and enhance clinical understanding of autoimmune GFAP-A.

## Methods

### Study subjects

Forty-three patients diagnosed with GFAP-A were retrieved from the information system of Tongji Hospital between March 2017 and July 2023. One patient was excluded due to a lack of CSF or serum GFAP-IgG test. Six patients who tested positive for GFAP-IgG in serum but negative in CSF were also excluded. Of the 36 patients who tested positive for GFAP-IgG in CSF, two were excluded due to a lack of imaging information. Overall, 34 patients were included in the final analysis. This retrospective observational study was approved by the institutional review board of Tongji Hospital and written informed consent was waived.

Demographics, clinical symptoms, previous history of tumor and autoimmune diseases, laboratory tests, and imaging findings of the medical records were retrospectively collected. Antibody detection was performed by Wuhan Kindstar Diagnostics Co., Ltd. (Wuhan, China) using a cell-based assay. The detected antibodies included GFAP, myelin oligodendrocyte glycoprotein (MOG), aquaporin 4 (AQP4), myelin basic protein (MBP), N-methyl-D-aspartate receptor (NMDAR), α-amino-3-hydroxy-5-methylisoxazole-4-propionic acid receptor (AMPAR1), AMPAR2, leucine-rich glioma inactivated 1 (LGI1), contactin-associated protein 2 (CASPR2), and γ-aminobutyric acid-B receptor (GABABR) in serum and CSF. Routine CSF analysis included intracranial pressure, total cell counts (neutrophils or lymphocytes), protein level, glucose level, chloride, IgG index, and number of oligoclonal bands (OB). Smears and cultures of CSF were performed to detect bacteria, *Mycobacterium tuberculosis*, and fungi. Additionally, CSF IgM antibody testing for various viruses including Epstein–Barr virus (EBV), herpes simplex virus (HSV)-1, HSV-2, varicella-zoster virus (VZV), cytomegalovirus (CMV), echovirus, parvovirus B19 (PVB19), coxsackievirus A16 (CA16), coxsackievirus B (CVB), measles virus (MV), and rhinovirus (RV) was carried out. For four cases, CSF metagenomic next-generation sequencing (mNGS) testing was performed. Concurrently, serum T-SPOT testing and serum IgM antibody testing for pathogens such as influenza A virus, influenza B virus, respiratory syncytial virus (RSV), *Mycoplasma pneumoniae*, and *Chlamydia pneumoniae* were conducted. Routine laboratory tests included measurement of serum electrolytes (Na+, Cl-), antinuclear antibodies, tumor markers, and OB. Electromyography (EMG) was performed if the patient presented with peripheral nerve symptoms and signs.

### Image acquisition and assessment

All MRI images were performed using 1.5 T or 3 T MRI scanners, including conventional T1-weighted image (T1WI), contrast-enhanced T1-weighted image (T1WI + C), T2-weighted image (T2WI), T2-weighted fluid-attenuated inversion recovery (T2/FLAIR) sequences, fat-suppressed fast spin-echo sequences, and diffusion-weighted image of the head, spinal cord, and optic nerve. Two radiologists (G.K and X.Z), with 8 and 12 years of experience in neuroradiology, respectively, reviewed all available MRI images independently to analyze the location, size, morphology, and enhancement features of all lesions. Discrepancies were resolved by discussion and consensus.

The brain was divided into the meninges, cerebral cortex, juxtacortical white matter, deep white matter, periventricular white matter, basal ganglia, thalamus, brainstem, and cerebellum. Spinal cord lesions longer than three vertebral segments were defined as longitudinal segment myelitis. The sagittal position was divided into cervical, thoracic, and lumbar (conical) segments. The transverse axis was classified into anterior, posterior, lateral, central, and transverse lesions. The optic nerve was divided into three sections: anterior, middle, and posterior segments.

### Statistical analysis

Data were expressed as frequencies and percentages for categorical variables and as median (range, minimum-maximum) for continuous variables. A paired samples t-test was used to compare the modified Rankin scale (mRS) score before and after treatment. Chi-squared test and a binary logistic regression analysis were used to analyze the correlation between higher mRS score, intensive care unit (ICU) admission rate and clinical or imaging manifestations.

## Results

### Demographic and clinical features

Overall, 34 patients diagnosed with GFAP-A were analyzed, of which 28 patients were males (82.4%). The median age at disease onset was 41.5 years (range 14–74 years). The median duration of the disease course was 20 days (range 3–210 days). Twenty patients (58.8%) presented with acute or subacute onset, while 14 patients (41.2%) presented with a progressive onset. The predominant clinical syndrome was meningoencephalomyelitis (13/34, 38.3%), followed by meningoencephalitis (12/34, 35.3%), encephalomyelitis (5/34, 14.7%), encephalitis (2/34, 5.9%), meningitis (1/34, 2.9%), and myelitis (1/34, 2.9%). Twenty-one patients (61.8%) had fever and 14 (41.2%) were initially diagnosed with intracranial infection. Twenty-one patients (61.8%) exhibited meningitis symptoms, with headache being the most common symptom (16/34, 47.1%). Twenty-three patients (67.6%) had encephalitis symptoms, with anxiety and depression (14/34, 41.2%) most frequent symptoms, followed by psychiatric symptoms (8/34, 23.5%). Fourteen patients (41.2%) had motor impairment, with tremor being the most common symptom (10/34, 29.4%). Seven patients (20.6%) presented with ataxia, 5 (14.7%) with blurred visions, and 4 (11.8%) with severe symptoms, such as loss of consciousness and cardiac and respiratory arrest. Other symptoms included decreased limb muscle strength (22/34, 64.7%), paresthesia (11/34, 32.4%), and autonomic function abnormalities (11/34, 32.4%). Clinical manifestations are shown in Table 1.

**Table 1:** Clinical symptoms of 34 patients with autoimmune GFAP astrocytopathy.

Clinical symptoms	Patients, No. (%)
Fever	21 (61.8%)
Meningitis	21 (61.8%)
Headache	16 (47.1%)
Meningeal sign (+)	15 (44.1%)
Nausea, vomiting	8 (23.5%)
Encephalitis	23 (67.6%)
Anxiety, depression	14 (41.2%)
Psychiatric symptoms	8 (23.5%)
Seizures	5 (14.7%)
Memory loss	4 (11.8%)
Dyskinesia	14 (41.2%)
Tremor	10 (29.4%)
Myoclonus	2 (5.9%)
Ataxia	7 (20.6%)
Blurred vision	5 (14.7%)
Loss of consciousness, breathing, and cardiac arrest	4 (11.8%)
Muscle weakness	22 (64.7%)
Hyperreflexia	15 (44.1%)
Pathological signs (+)	13 (38.2%)
Abnormal sensation	11 (32.4%)
Autonomic dysfunction	11 (32.4%)
Bladder dysfunction	9 (26.5%)
Abnormal intestinal function	4 (11.8%)

### Laboratory examination

Thirteen patients (38.2%) tested positive for GFAP-IgG in both CSF and serum, and 21 (61.8%) tested negative for GFAP-IgG in serum. Five patients (14.7%) had coexisting neural autoantibodies: AQP4-IgG (*n* = 1), ganglioside-monosialic acid (GM1)-IgG (*n* = 1), NMDAR-IgG (*n* = 2), and dipeptidyl-peptidase-protein 6 (DPPX)-IgG (*n* = 1). Three patients (8.8%) had coexisting autoimmune thyroiditis and one patient (2.9%) had coexisting ovarian teratoma. An elevated DNA copy number of EBV was detected in the CSF of two patients (5.9%). One patient (2.9%) tested positive for influenza virus type A IgM antibody and another patient tested positive for VZV IgM antibody.

Serology and CSF results are presented in Table 2. One-half of the patients (50%) had hyponatremia and 12 (35.3%) had hypochloraemia. Six patients (17.6%) had increased intracranial pressure, confirmed by lumbar puncture. Twenty-six patients (76.5%) had elevated CSF cell count (median 45 × 10^6^/L, range 0–1,600 × 10^6^/L), of which 15 had lymphocytic pleocytosis. Twenty-seven patients (79.4%) had elevated protein levels (median 940 mg/L, range 180–5,713 mg/L), 5 (14.7%) had decreased glucose levels, and 16 (47.1%) had decreased chloride levels. The IgG index increased in 10 cases (58.8%). Seven patients (31.8%) tested positive for OB in CSF and negative for OB in serum.

**Table 2:** CSF and serum findings of 34 patients with autoimmune GFAP astrocytopathy.

Laboratory and serologic Findings	Patients, No. (%)	Median [range]
CSF examination		
Intracranial hypertension (>180mmH_2_O)	6 (17.6)	
Nucleated cell count (10^6^/L)		45 (0–1,600)
Elevated nucleated cell count (>5 × 10^6^/L)	26 (76.5)	
Lymphocyte proportions		90 (80–96)
Lymphocytic pleocytosis (>70%)	15/15 (100)	
Protein level (mg/L)		940 (180–5,713)
Protein elevation(>450 mg/L)	27 (79.4)	
Glucose (mmol/L)		3.24 (1.60–4.61)
Decreased glucose (<2.25 mmol/L)	5 (14.7)	
Chloride (120-130 mmol/L)		120 (102–130)
Decreased chloride (<120 mmol/L)	16 (47.1)	
IgG index		0.9 (0.6–1.3)
Elevated IgG index (>0.7)	10/17 (58.8)	
GFAP-IgG (+)	34 (100)	
Serum findings		
GFAP-IgG (+)	13 (38.2)	
Sodium (mmol/L)		135 (121–141)
Hyponatremia (<135 mmol/L)	17 (50)	
Chloride (mmol/L)		97.3 (85.4–106)
Hypochloraemia (<96 mmol/L)	12 (35.3)	
CSF OB (+), serum OB (−)	7/22 (31.8)	

### MRI features

**Figure 1:**
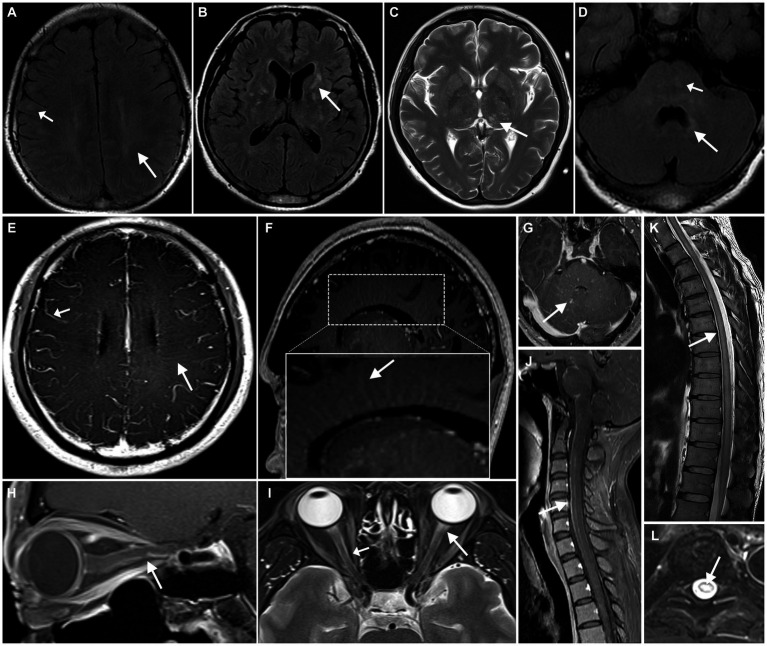
Neuroimaging findings with autoimmune GFAP astrocytopathy. cerebral parenchymal lesions can be observed in the subcortical white matter (**A**, short arrow), periventricular regions (**A**, arrow), basal ganglia **(B)**, thalamus **(C)**, brainstem (**D**, short arrow), and periventricular regions of the fourth ventricle (**D**, arrow). MRI enhancement shows enhancement of the leptomeninges (**E**, short arrow) and radial linear enhancement surrounding the lateral ventricles (**E,F**, arrow) and fourth ventricle **(G)**. A hyperintense lesion is observed in the anterior portion of the optic nerve on T2WI (**I**, arrow), with the optic nerve sheath showing hyperintensity on T2WI (**I**, short arrow) and enhancement on contrast-enhanced MRI **(H)**. Spinal cord shows longitudinally extensive transverse myelitis **(K,L)**, with linear enhancement seen in the meninges and central canal of the spinal cord **(J)**.

Of the 33 patients with plain MRI (Table 3; Figure 1), 30 (90.9%) had abnormal hyperintense lesions on T2WI and T2/FLAIR images. The lesions were frequent in juxtacortical white matter (18/33, 54.5%), followed by periventricular white matter (16/33, 48.5%), basal ganglia (15/33, 45.5%), brainstem (11/33, 33.3%). Among the patients with brainstem lesions, 4 (36.4%) experienced cardiac arrest or respiratory arrest. Nine patients (27.3%) had thalamic lesions and 2 (6.1%) had cerebellar lesions. Two patients (6.1%) had area postrema lesions, with no intractable hiccups. On gadolinium-enhanced brain MRI (Table 3; Figure 1), 24 of 33 patients (72.7%) had abnormal brain enhancement, with supratentorial leptomeningeal enhancement being the most frequent (15/33, 45.5%), followed by linear perivascular radial gadolinium enhancement (14/33,42.4%).

**Table 3:** Neuroimaging findings of brain and orbital of 34 patients with autoimmune GFAP astrocytopathy.

MRI findings	Patients, No.(%)
Brain MRI (*n* = 33)	
Juxtacortical white matter	18 (54.5)
Periventricular white matter	16 (48.5)
Basal ganglia	15 (45.5)
Brainstem	11 (33.3)
Thalamus	9 (27.3)
Deep white matter	9 (27.3)
Cortex	4 (12.1)
cerebellum	2 (6.1)
Gadolinium-enhanced brain MRI (*n* = 33)	
Supratentorial leptomeningeal enhancement	15 (45.5)
Infratentorial leptomeningeal enhancement	11 (33.3)
Linear perivascular radial enhancement around ventricle	14 (42.4)
Linear perivascular radial enhancement around the fourth ventricle	3 (9.1)
Intraparenchymal enhancement	9 (27.3)
Orbital MRI (*n* = 7)	
Optic nerve sheath edema	5 (71.4)
Anterior part of optic nerve	3 (42.9)
Orbital MRI enhancement (*n* = 4)	
Optic nerve sheath enhancement	4 (100)
Anterior part of optic nerve enhancement	1 (25.0)

Five of 7 patients (71.4%) who underwent an orbital MRI scan exhibited optic nerve sheath edema (Table 3; Figure 1). All 4 patients (100%) who underwent orbital MRI enhancement had optic nerve sheath enhancement (Table 3; Figure 1).

Nineteen of 27 patients (70.4%) who underwent spine MRI exhibited hyperintense intramedullary lesions on T2WI. Long segments (15/27, 55.6%) and transverse lesions (14/27, 51.9%) were the most common lesions. The thoracic spine was the most common location (7/27, 25.9%), followed by the total length of the spinal cord (6/27, 22.2%).

Thirteen of 22 patients (59.1%) who underwent enhanced spine MRI had abnormal spinal cord enhancement (Table 4; Figure 1). Leptomeningeal enhancement was the most frequent enhancement pattern (12/22, 54.5%), followed by spinal central canal enhancement (8/22, 36.4%).

**Table 4:** Neuroimaging findings of spine of 34 patients with autoimmune GFAP astrocytopathy.

MRI findings	Patients, No. (%)
Spine MRI (*n* = 27)	
Lesion pattern of spine in the sagittal position	
≥ 3 vertebra segments	15 (55.6)
<3 vertebra segments	4 (14.8)
Lesion location of spine in the sagittal position	
Thoracic segment	7 (25.9)
Cervical, thoracic, and lumbar (conical) segment	6 (22.2)
Cervical and thoracic segment	5 (18.5)
Thoracic and lumbar segment	1 (3.7)
Cervical segment	0 (0)
Lumbar (conical) segment	0 (0)
Lesion location of spine in the transversel position	
Transverse	14 (51.9)
Lateral	4 (14.8)
Anterior	4 (14.8)
Posterior	3 (11.1)
Central	1 (3.7)
Gadolinium-enhanced spine MRI (*n* = 22)	
Leptomeningeal enhancement	12 (54.5)
Spinal central canal enhancement	8 (36.4)
Intramedullary enhancement	9 (40.9)

### Other examinations

Of the 34 patients, 4 (11.8%) had peripheral neuropathy confirmed by EMG: 2 with motor nerve fiber axonal neuropathy of the lower extremities, 1 with motor nerve fiber axonal neuropathy of the lower and upper extremities, and 1 with sensory nerve fiber axonal neuropathy of the upper extremities. Additionally, 2 (5.9%) had bilateral visual pathway conduction issues through visual-evoked potentials, and 1 (2.9%) had papilledema confirmed by fundus examination.

### Treatment and prognosis

After a median follow-up time of 57.5 days (range 11–1,197 days), 29 of 34 patients (85.3%) had varying degrees of symptom improvement. Glucocorticoid treatment improved symptoms in 13 of 15 patients (86.6%). In addition, a combination of intravenous methylprednisolone (IVMP) and intravenous immunoglobulin (IVIG) treatment improved symptoms in 5 of 6 patients (83.3%). Furthermore, 8 of 9 patients (88.9%) improved by combining IVMP with tacrolimus. A combination of IVMP, IVIG, and tacrolimus also improved symptoms in 3 of 4 patients (75.0%). Importantly, the rates of symptom improvement were not significantly different between the four treatments (*p* = 0.925). Three of 34 patients (8.8%)had relapses during follow-up. However, patients’ symptoms improved after re-treatment with IVMP, IVIG, and tacrolimus.

**Figure 2:**
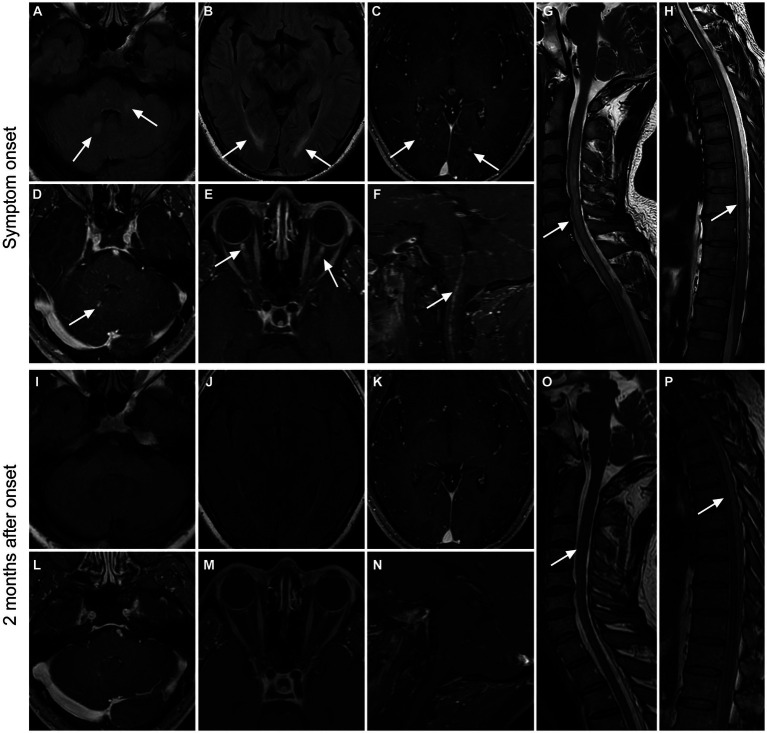
Imaging findings in a patient with GFAP. A 36-year-old male presented with bilateral lower limb weakness and numbness, accompanied by unsteady gait, with a subacute onset. After treatment with glucocorticoids, intravenous immunoglobulin, and tacrolimus, there was an improvement in symptoms, and a follow-up examination after 2 months showed a mRS score of 1. Initial MRI examination during the onset of the disease **(A–H)** revealed high T2WI signal in the cerebellar hemisphere and brainstem **(A)**, as well as in the periventricular white matter surrounding the bilateral lateral ventricles **(B)**. Additionally, there were punctate enhancements observed in the periventricular regions surrounding the bilateral lateral ventricles (**C**, arrows), as well as in the cerebellar hemisphere and brainstem (**D**, arrow). Furthermore, there was prominent enhancement in the anterior-middle portion of the bilateral optic nerves **(E)** and linear enhancement in the central canal of the medulla oblongata and cervical spinal cord **(F)**. The cervical spinal cord **(G)**, thoracic cord, and conus medullaris **(H)** had hyperintensity on T2WI throughout the entire segments. Follow-up MRI examination at 2 months after onset **(I–P)** showed the disappearance of T2WI hyperintensity and enhanced lesions in the brain parenchyma **(I–L)**. The enhancements in the optic nerves and medulla oblongata-cervical cord central aqueduct disappeared **(M,N)**. Most of the T2-weighted hyperintensity lesions in the spinal cord were resolved **(O,P)**.

Thirteen patients received imaging follow-up, with a median follow-up time of 46 days (range: 11–477 days). As shown in Table 5; Figure 2, 7 of 11 patients (63.6%) had decreased cerebral parenchymal lesions, while 3 of 4 patients (75%) had decreased cerebral parenchymal enhancement. In addition, meningeal enhancement decreased in all 4 cases (100%), and perivascular radiation enhancement decreased in all 3 cases (100%). Spinal cord lesions decreased in all 7 cases (100%), with 5 cases (100%) showing decreased spinal cord lesion enhancement. Additionally, meningeal and central canal enhancement was reduced or disappeared in all 4 cases (100%). For optic nerve lesions, 2 cases (100%) had a decrease in size, and 1 case (100%) had a decrease in both optic nerve lesions and sheath enhancement.

**Table 5:** Changes in imaging findings after treatment and follow-up.

	Improvement	No improvement	Follow-up time (days)
Imaging manifestations			
Brain parenchymal lesion	7	4	39 (16–81)
Enhancement of brain parenchymal lesions	3	1	44 (34–85)
Meningeal enhancement.	4	0	39 (11–133)
Linear perivascular radial enhancement	3	0	43 (38–133)
Intramedullary lesions	7	0	39 (17–477)
Enhancement of intramedullary lesions	5	0	34 (17–41)
Enhancement of the meninges and central canal of the spinal cord.	4	0	30.5 (17–41)
Optic nerve lesions	2	0	267 (72–461)
Enhancement of the lesion of the optic nerve and the optic nerve sheath	1	0	43

IVMP, intravenous methylprednisolone; IVIG, intravenous immunoglobulin.

The prognosis was assessed using the mRS score. An mRS score of 0–2 was considered a good prognosis, whereas a score > 2 was considered a poor prognosis. The median onset mRS score was 2.0 (range 0–5). At the last follow-up, the median mRS score was 1.0 (range 0–4), with a median follow-up time of 57.5 days (range 11–1,197 days). There was a statistically significant difference in the mRS score before and after treatment (*p* < 0.001).

The potential factors influencing the rate of ICUadmission and last follow-up mRS score were assessed (Table 6). The factors included in the analysis were age, sex, hyponatremia, meningitis, linear perivascular radial enhancement, basal ganglia, thalamic, brainstem, and intramedullary lesions. The results indicated a possible positive association between brainstem lesions and the rate of ICU admission (*p* < 0.05). Additionally, brainstem lesions (*p* < 0.05) were positive associated with a higher onset mRS score. Linear perivascular radial enhancement was also positive associated with poor prognosis. However, the use of IVIG did not significantly improve prognosis (*p* > 0.05).

**Table 6:** Correlation between higher mRS score, ICU admission and clinical or imaging manifestations.

	ICU admission	P	Onset mRS score (3–6)	P	Last follow-up mRS score (3–6)	P
	(*n* = 6)		(*n* = 11)		(*n* = 3)	
Age (>50y), *n* = 12	1	0.293	4	0.928	1	0.941
Gender (male), *n* = 28	5	0.945	9	0.955	2	0.455
Hyponatraemia, *n* = 17	1	0.072	5	0.714	3	0.07
Meningitis, *n* = 25	5	0.549	8	0.942	3	0.276
Linear perivascular radial enhancement, *n* = 14	3	0.628	6	0.273	3	0.03
Basal ganglia lesions, *n* = 15	4	0.22	6	0.397	1	0.694
Thalamic lesions, *n* = 9	3	0.15	4	0.366	1	0.778
Brainstem lesions, *n* = 11	4	0.048	7	0.007	2	0.183
Intramedullary lesions, *n* = 19	5	0.196	9	0.064	3	0.238
IVIG therapy, *n* = 10			5	0.156	2	0.138

IVIG, intravenous immunoglobulin.

## Discussion

The present study retrospectively analyzed the clinical and MRI features of 34 GFAP-A patients. The study cohort was predominated by male patients, accounting for 82.4% of the total sample. The most common presentation was meningoencephalomyelitis, followed by meningoencephalitis, which is consistent with findings from previous reports. The lesions on the brain MRI were frequent in juxtacortical white matter, followed by periventricular white matter, basal ganglia, brainstem, and thalamus. Most patients (70.4%) had hyperintense intramedullary lesions, and long segments and transverse lesions were the most common. The thoracic spine was the most frequently involved, followed by the total length of the spinal cord. Leptomeningeal enhancement was the most common enhancement pattern, followed by linear perivascular radial enhancement, which may lead to a high misdiagnosis rate of intracranial infection in the early disease course. These findings may provide new insight into the differential diagnosis of GFAP-A.

A majority of the enrolled patients were males, with a male: female ratio of 4.7:1. Although differences in male gender susceptibility were observed between our study and a pooled analysis (male: female ratio = 1.01:1) (8), this may be not significant owing to the small sample sizes of our cohort.

Detection of GFAP-IgG in CSF is a biomarker of GFAP-A. Overall, 34 patients tested positive for GFAP-IgG in CSF, of which the majority (61.8%, 21/34) exhibited negative serum GFAP-IgG. In addition, 58.8% of patients had an increased IgG index in CSF, and 31.8% presented with a positive OB in CSF and a negative OB in serum. It was suggested that GFAP-IgG may be synthesized in the brain (9). The biopsies of GFAP-A patients revealed that CD138^+^ cells — secreting high levels of antibodies — were present in the interstitial and perivascular spaces. This explained the intrathecal synthesis of antibodies and higher titers of pattern-specific antibodies in the CSF than in the serum (9).

The production of GFAP-IgG in patients with GFAP-A may be a result of the virus triggering GFAP autoimmunity or the viral invasion of astrocytes exposing GFAP expression. Our data showed that 11.8% of the patients presented with a viral infection and 61.8% presented with fever. It is also possible that certain autoimmune diseases initiate primary inflammation and disrupt astrocytic function, whereas GFAP autoimmunity is associated with the secondary phenomenon (5). For instance, it was found that two patients tested positive for NMDAR-IgG, one for AQP 4-IgG, and one for DPPX-IgG.

One patient had ovarian teratoma, which was the most frequently coexisting tumor of GFAP-*A. fang* et al. found that more than one-third of GFAP-A is associated with tumors, and the occurrence of GFAP-A may be related to the paraneoplastic mechanism (4). Immunohistochemical staining of ovarian teratomas from GFAP-A patients revealed that the cytoplasm of glial processes in neuronal tissue and epithelial cells reacts strongly with GFAP-IgG and ectopic expression of this nervous system tissue in tumors trigger the immune response (10). GFAP-IgG is considered non-pathogenic and does not directly interact with intracellular antigen GFAP, while GFAP-specific cytotoxic T cells play a central role in the immune response (11).

About 61.8% of the patients had meningitis symptoms or signs and 76.5% had inflammatory changes in CSF. Imaging results showed that 45.5 and 54.5% of the patients had leptomeningeal enhancement of the brain and spine, respectively, 42.4% had linear perivascular radial enhancement, and 36.4% had spinal central canal enhancement. The immunofluorescence pattern of patient IgG binding to rodent CNS tissues shares similarities with the brain and spinal cord MRI patterns seen in patients diagnosed with GFAP-A (4). The main pathological characteristic is the presence of CD8^+^ T lymphocytic infiltration in the white matter, mainly perivascular (4, 12, 13). Previous studies found that leptomeningeal enhancement was the predominant feature in the early stage of GFAP-A and may precede the radiographic appearance of myelitis (14, 15). Thus, meningitis symptoms or signs, inflammatory changes in CSF, and meningeal enhancement on MRI made it difficult to differentiate GFAP-A from intracranial infection, which also led to the initial diagnosis of intracranial infection in 41.2% of patients in the present study. Distinguishing between GFAP-A and intracranial viral infections presents a significant challenge, as viruses can potentially trigger GFAP-A and coexist with it. Common viruses linked to GFAP-A include EBV, VZV, and HSV (16). Meningitis is rare in EBV, VZV, and HSV infections (17), suggesting it may be due to an autoimmune response. While acyclovir effectively treats encephalitis caused by these viruses, its efficacy is suboptimal when GFAP is involved with EBV. Conversely, corticosteroid treatment has shown considerable effectiveness (18). Recent reports have described cases where GFAP-A mimics CNS tuberculosis, characterized by lower chloride levels and CSF glucose to serum glucose ratios (19, 20). In our study, one case initially suspected to be tuberculosis meningitis had ineffective diagnostic anti-tuberculosis treatment. Subsequent detection of positive GFAP-IgG in the CSF, along with symptom improvement and imaging findings after corticosteroid and IVIG treatment, led to the final diagnosis of GFAP-A. Therefore, for cases with an initial diagnosis of meningitis or meningoencephalitis, the possibility of GFAP-A should be considered to reduce the rate of misdiagnosis. Meanwhile, it has been reported that brain linear perivascular radial gadolinium enhancement is a hallmark of GFAP-A, which was frequent in 53% of patients in a case series of 102 patients (5) and 42.4% of patients in the present study, but slightly less frequent than leptomeningeal enhancement. However, such radial perivascular enhancement pattern was also observed in other diseases with negative GFAP-IgG, such as peripheral lymphoma, meningoencephalitis, and coronavirus disease 2019-associated acute disseminated encephalomyelitis (21, 22). Although these studies reported a low positive rate (0.92%, 5/541), caution should be exercised in utilizing this imaging for the diagnosis of GFAP-A in individuals with clinically suspected encephalitis. Otherwise, it can lead to misdiagnosis in patients with other diseases such as tumors.

Further, brain parenchyma lesions had obvious heterogeneity, with encephalitis symptoms appearing in 67.6% of the cases, mainly presenting as anxiety, depression, and mental disorders. The clinical presentation of GFAP-A encephalitis and the MRI findings of brain cortex lesions resemble those of viral encephalitis and other autoimmune encephalitis. Additionally, in GFAP-A, lesions can also occur in the basal ganglia, thalamus, brainstem, and cerebellum. These lesions were spot-like, with iso-or slightly hyper-T2WI signal, indistinct boundary, and perivascular punctate enhancement. Although common demyelinating lesions such as optic neuromyelitis spectrum disease (NMOSD) and multiple sclerosis (MS) can involve the brain stem and cerebellum, they rarely involve the basal ganglia and thalamus. Kimura et al. suggested that the bilateral hyperintensities of the posterior part of the thalamus characterize GFAP-A (23). In their case series, 6 patients (6/14, 43%) presented with abnormal hyperintense lesions on T2WI/FLAIR in the thalamus. In our case series, 9 patients (9/33, 27.3%) had abnormal changes in the thalamus, which was the fifth common location of the brain parenchyma. The other four locations were juxtacortical white matter (18/33, 54.5%), periventricular white matter (16/33, 48.5%), basal ganglia (15/33, 45.5%), and brainstem (11/33, 33.3%).

Approximately 70.4% (19/27) of patients undergoing spinal MRI had myelitis. It frequently presented as longitudinally extensive lesions and was often located in the thoracic or whole spinal cord segment. Spine lesions were often presented with a slightly hyper-T2WI signal and indistinct boundary. It was distinct from lesions typically encountered in NMOSDs, characterized by a higher T2WI signal and bright spotty lesions (24). Meanwhile, GFAP-A myelitis enhancement often had punctate enhancement, which can also be differentiated from open ring enhancement in MS.

Most patients had a mild to moderate increase in CSF pressure in previous studies (4, 25–27) but the proportion was not high in the current study. Only 6 cases (6/34, 17.6%) had intracranial hypertension. The pathophysiology of intracranial hypertension in autoimmune encephalitis remains unknown. It may be that inflammatory contents (such as lymphocytes and cytokines) disrupt CSF reabsorption by arachnoid granulations either through mechanical obstruction or toxic effects (28). Intracranial hypertension can also lead to optic disc edema and optic nerve sheath edema.It is also possible that these alterations result from an autoimmune reaction against GFAP, which is expressed in the retina by astrocytes and Müller cells, particularly in the peripapillary region. It may also explain the enhancement of the sheath membrane, often involving the anterior part of the optic nerve.

Hyponatremia was observed in half of the cases in our study; however, this was rarely observed in other demyelinating or autoimmune encephalitis, possibly due to cerebral salt depletion syndrome or inappropriate antidiuretic hormone secretion syndrome (23). About 6% (2/34) of patients in the present cohort had lesions in the postrema area but no related symptoms were observed. The rate of area postrema syndrome (APS) was lower in our case series than in previous reports. A precious study found that 11% of GFAP-A had lesions in the postrema area, with hiccup being the predominant symptom (26). APS is one of the core symptoms of NMOSD (29). Although the incidence of APS in GFAP-A was relatively lower than that in NMOSD, APS is not uncommon in GFAP-A and may fail to discriminate this disorder from NMOSD (26).

GFAP can be expressed in Schwann cells, dorsal root, and cranial nerve ganglia (especially the trigeminal nerve). Some studies have reported that GFAP-A can combine with axonal large fiber polyneuropathy, cranial nerve, and spinal nerve root lesions (30, 31). The EMG results revealed that 4 patients (11.8%) developed axonal neuropathy. Other research studies have also indicated that the major phenotype of GFAP-related peripheral neuropathy primarily manifests as axonal injury, specifically affecting the motor fibers in the lower extremities (32). Interestingly, GFAP, an intracellular protein, is not expressed in axons. Furthermore, findings from peripheral nerve biopsies demonstrated the presence of perivascular inflammatory infiltrates predominantly composed of T cells. The precise role of GFAP-IgG in the development of peripheral nerve axonal damage and the underlying mechanisms remain unclear that require further exploration and investigation.

Previous studies found that the majority of patients responded to immunotherapy (10, 30). Our study found that 85.3% of patients responded to immunotherapy, with improvements in both symptoms and imaging findings, as well as a reduction in the mRS score. While some studies have suggested that combining IVMP with IVIG is more effective than IVMP alone (25), others have reported that patients receiving a high dose of IVMP alone or in combination with IVIG or plasma exchange had similar initial clinical responses without significant differences in clinical improvement after immunotherapy (8). Similarly, we found no significant advantage of IVIG or tacrolimus combined with IVMP over IVMP alone. Hence, larger trials are needed to reach a definitive conclusion. Patient prognosis was good, with a median mRS score of 1 after a median follow-up of 57.3 days, consistent with the findings of a previous study (5).

There was a positive correlation between a more severe onset of disability (a higher mRS score) and a higher rate of ICU admission in patients with lesions in the brainstem. This may be because these anatomical regions are closely associated with motor function. The brainstem — particularly the medulla — plays a critical role in respiratory and circulatory function, predisposing patients to respiratory failure and cardiac arrest. Therefore, active management of patients with brain stem lesions is critical. Additionally, we found that cases with linear perivascular radial enhancement had a poorer prognosis. This could be due to the increased severity of the inflammatory response and the greater extent of the lesions, leading to more extensive nerve damage. However, it is noteworthy that studies exploring the factors that influence prognosis are scanty; thus, further large, multicenter studies are warranted to validate these findings.

Nonetheless, this study has several limitations. Firstly, this is a retrospective cross-sectional study, and no case–control and cohort studies were performed; however, the initial visit case data were relatively complete. Secondly, since all cases were from a single center, there may be selection bias. Thirdly, some cases were referred to our hospital for imaging examination after a period of treatment, which may lead to a low positive rate of some imaging findings. Therefore, studies with larger sample sizes and long-term follow-up are necessary to develop standard treatment regimens for GFAP-A and validate our findings.

In conclusion, the current study demonstrated that GFAP-A often presented with meningoencephalomyelitis and meningoencephalitis. The lesions were often located in the juxtacortical white matter, periventricular white matter, basal ganglia, brainstem, and thalamus of the brain. Spine lesions were often long segments and transverse, often involving the thoracic spine and the total length of the spinal cord. Leptomeningeal enhancement was the most common enhancement pattern, followed by linear perivascular radial enhancement. Overall, the study findings may provide new insight into the differential diagnosis of GFAP-A.

## Data availability statement

The raw data supporting the conclusions of this article will be made available by the authors, without undue reservation.

## Ethics statement

The studies involving humans were approved by the institutional review board of Tongji Hospital. The studies were conducted in accordance with the local legislation and institutional requirements. Written informed consent for participation was not required from the participants or the participants’ legal guardians/next of kin in accordance with the national legislation and institutional requirements.

## Author contributions

GK: Writing – original draft, Writing – review & editing. SJ: Data curation, Formal analysis, Investigation, Writing – review & editing. TY: Data curation, Writing – original draft. XZ: Conceptualization, Data curation, Formal analysis, Funding acquisition, Investigation, Methodology, Project administration, Software, Supervision, Validation, Visualization, Writing – original draft, Writing – review & editing.
